# Menisci protect chondrocytes from load-induced injury

**DOI:** 10.1038/s41598-018-32503-1

**Published:** 2018-09-20

**Authors:** Z. Abusara, S. H. J. Andrews, M. Von Kossel, W. Herzog

**Affiliations:** Human Performance Laboratory, Faculty of Kinesiology, University of Calgary, Alberta, Canada

**Keywords:** Cellular imaging, Chondrocytes, Cartilage, Skeletal muscle, Nonlinear optics

## Abstract

Menisci in the knee joint are thought to provide stability, increased contact area, decreased contact pressures, and offer protection to the underlying articular cartilage and bone during joint loading. Meniscal loss or injury is typically accompanied by degenerative changes in the knee, leading to an increased risk for osteoarthritis in animals including humans. However, the detailed mechanisms underlying joint degeneration and the development of osteoarthritis remain largely unknown, and the acute effects of meniscal loss have not been studied systematically. We developed a microscopy-based system to study microscale joint mechanics in living mice loaded by controlled muscular contractions. Here, we show how meniscal loss is associated with rapid chondrocyte death (necrosis) in articular cartilage within hours of injury, and how intact menisci protect chondrocytes *in vivo* in the presence of intense muscle-based joint loading and/or injury to the articular cartilage. Our findings suggest that loading the knee after meniscal loss is associated with extensive cell death in intact and injured knees, and that early treatment interventions should be aimed at preventing chondrocyte death.

## Introduction

Osteoarthritis (OA) is a degenerative joint disease, primarily characterized by the destruction of articular cartilage^1^. Articular cartilage is a thin (2–4 mm in human joints^2^) layer of fibrous connective tissue covering the articulating surfaces of bones in synovial joints. Arthritic diseases are a leading cause of disability worldwide with approximately 9 million people affected in the United States alone^3^. Osteoarthritis has been associated with increased mortality, likely due to reduced mobility, and increased rate of comorbidities, such as cardiovascular disease and obesity^4^. Articular cartilage contains cells (chondrocytes) that occupy 2–15% of the volume, and an extracellular matrix (85–98% of total volumetric fraction) of which 65–80% is water^5^. Articular cartilage is a viscoelastic, biphasic material^6^ that, in conjunction with the synovial (joint) fluid, allows for virtually frictionless movement of the joint surfaces^7,8^.

Recently, we developed methods to quantify joint, bone, cartilage and chondrocyte mechanics in the intact knee of live mice, loaded by controlled muscular contractions^9–11^. Depending on the magnitude and duration of muscular loading, we demonstrated that chondrocyte death can result in the fully intact joint, under physiological muscle loading conditions^12^, and that joints show histological signs of onset of OA when exposed to prolonged loading^13^. Therefore, it appears that loading provided exclusively by muscles surrounding a healthy, intact joint, can trigger the onset, and potentially accelerate the rate of progression, of OA. However, the mechanisms underlying these events remain unknown^14^.

The menisci are fibrocartilaginous tissues in the knee whose primary roles are thought to include protection of the articular cartilage and stabilization of the joint^15^. The protective role of the menisci has been illustrated repeatedly in epidemiologic and experimental studies^16,17^, with a 14-fold increase in the risk of OA development after meniscectomy^16^. Fairbank described the association between meniscectomy and the development of OA some 70 years ago^18^. However, the detailed mechanisms by which meniscal loss puts the knee at risk of OA have yet to be elucidated. Recently, the protective role of the menisci has become a focus of investigation by the emergence of the destabilized medial meniscus (DMM) model as a reliable method of inducing OA in the knees of rodents^17,19^. Mechanically, the menisci are thought to reduce contact stresses and strains on the tibial plateau and play a role in governing fluid flow in articular cartilage^20–23^. Computer modeling suggests that the initial loading of the menisci, prior to cartilage-cartilage contact, may increase fluid pressure in articular cartilage, stiffening the tissue, and protecting chondrocytes from injurious strains^24^. However, experimental evidence of the role of the menisci in protecting joints from wear and tear remains scarce, non-systematic, and qualitative.

The objective of this study was to evaluate the protective role of the menisci and systematically quantify the possible mechanism(s) by which menisci reduce the risk of OA onset and progression. We used an *in vivo* murine knee model of focal cartilage lesions subjected to physiological muscle loading conditions with and without medial meniscectomy to address this objective.

## Materials and Methods

### Animal preparation

This study was carried out in accordance with the guidelines of the Canadian Council on Animal Care and was approved by the committee for Animal Use and Ethics at the University of Calgary.

**Figure 1:**
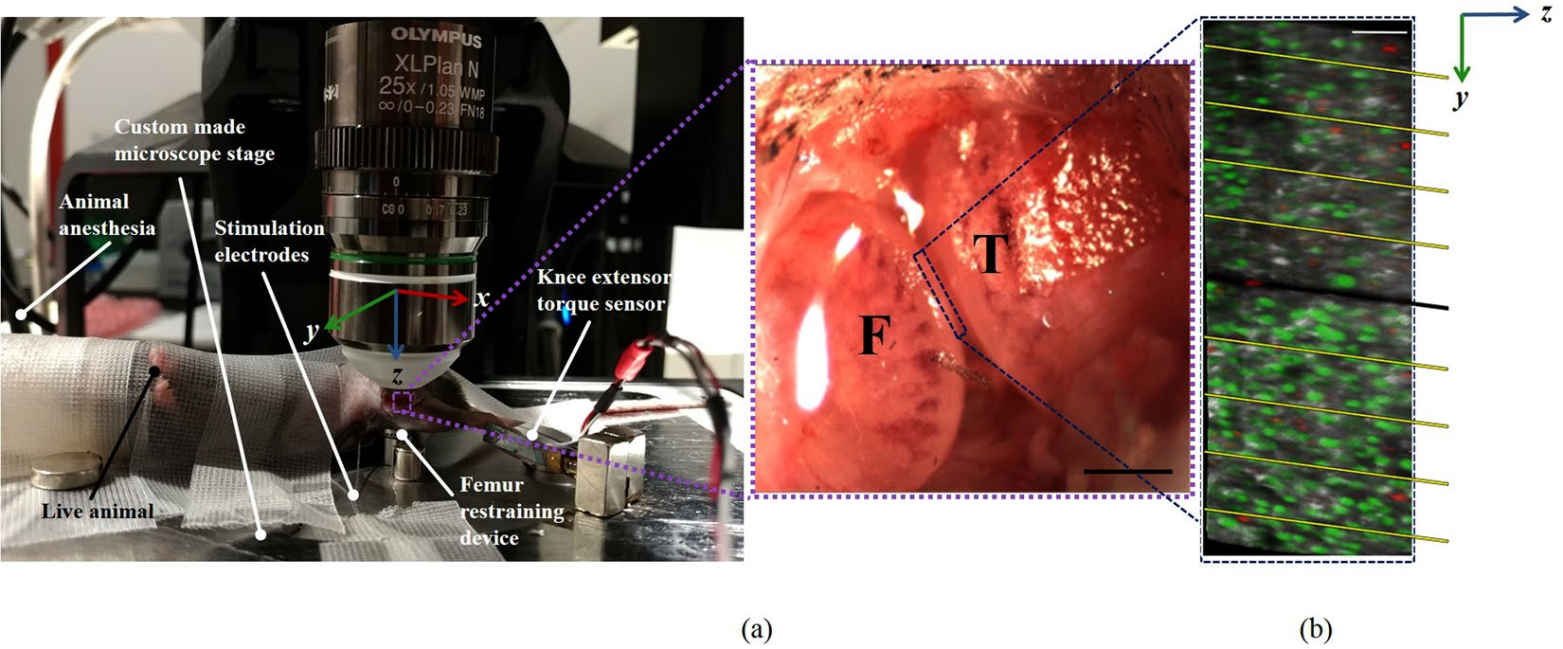
(**a**) Exposed mouse knee preparation showing the medial tibial plateau (T), and the medial femoral condyle (F) with the meniscus removed. Scale bar = 1000 µm. (**b**) 3D image of the cartilage showing the injury (black middle line, ~20 µm width, ~350 µm length) and the zonal sections used to count the cell death. Scale bar = 100 µm and the spacing between yellow lines = 100 µm.

Thirty-nine adult, male mice (10–12 weeks of age) were used in this study. Mice were anesthetized with an isoflurane/oxygen mixture (1–3%). The right knee joint was shaved and secured in a stereo-tactic frame that was rigidly attached to the stage of a dissecting microscope. The medial aspect of the joint was exposed with a 6 mm incision just posterior to the medial collateral ligament (Fig. 1a).

**Figure 2:**
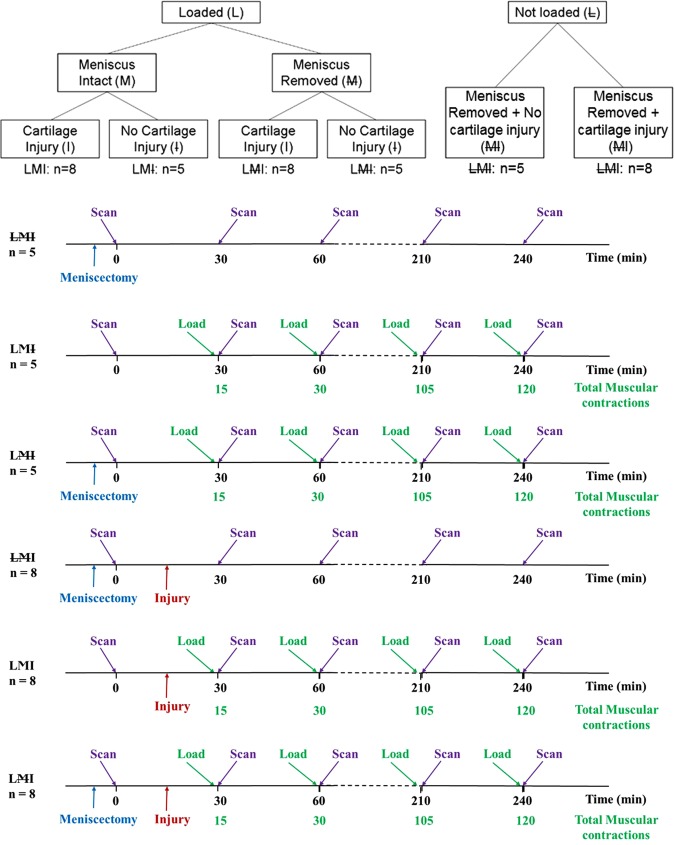
Flow charts illustrating the experimental groups.

Mice were divided into 6 experimental groups based on whether they were loaded by muscular contraction or not (i.e. 4 loaded groups and 2 non-loaded control groups). The loaded groups were divided by whether their meniscus was left intact or was removed, and whether they received a cartilage injury or not (Fig. 2). The cartilage injury (scalpel cut ~20 µm width, ~350 µm length) was applied underneath the medial meniscus and across the cartilage in the cartilage contact region, as shown in Fig. 1. The experimental groups were identified by the following notation: loaded (L), unloaded (L) meniscus intact (M), meniscus removed (M) and cartilage injury (I) or no cartilage injury (I). Three animals were chosen at random from LMI, LMI and LMI groups to quantify apoptotic vs. necrotic cell deaths.

### Cell viability staining

The exposed medial aspect of the knee was washed and filled with 30 µl of prepared Calcein AM and Ethidium homodimer-1 for live and dead cell identification respectively (Molecular Probes/ Invitrogen, USA). After a 30 minute incubation period in complete darkness, excess stain was removed and the area was washed and filled with a fresh phosphate buffered saline solution (PBS) allowing for the use of a water-immersion objective.

### Multi-photon microscopy and second harmonic generation (SHG)

Following staining, anesthetized mice were moved onto the stage of a multi-photon microscope (FVMPE-RS, Olympus, Japan). Medial femoral condyle cartilage was imaged using a 25 × 1.05 NA water-immersion objective (Olympus Inc., Japan) coupled with two independent multi-photon infrared pulsed lasers (InSight DS and Mai Tai DeepSee, Spectra Physics Inc., USA) enabling simultaneous excitation at different wavelengths. The first laser was tuned to 800 nm to produce SHG while the second laser was tuned to 940 nm to excite both live and dead cell stains. The emission signals were directed to a single-edge dichroic beam splitter (FF458-Di02, Semrock inc., USA) to separate the SHG signal from the live/dead cell signal. Live and dead cell signals were further separated using a dichroic beam splitter (FF570-Di01, Semrock inc. USA) and were then focused onto two non-descanned detectors through two single bandpass filters, FF01-520/35 and FF01-612/69 (Semrock inc., USA) to capture the live and dead cell signals respectively. The SHG signal was directed to a single-band bandpass filter centered at 400 nm (FF01-400/40, Semrock inc., USA) prior to focussing it onto a sensitive GaAsp non-descanned detector.

### Muscular loading of the mouse knee

Controlled muscular loading of the knee was achieved by stimulation of the knee extensor muscles using two fine wire electrodes inserted into the quadriceps muscle group. Muscles were stimulated using electrical stimulation with a Grass (S8800) digital stimulator, as we have previously described^10,11,25^. We have also demonstrated that a minimum load of 50% of the maximal muscular contraction is required to establish contact between the two cartilage surfaces^11^, so we aimed for 80% muscular load which is within the physiological range and leads to clear cartilage to cartilage contact and deformation when the medial meniscus is removed. The free tips of the exposed fine wires were separated by 2 mm, and application of approximately 7 volts at a frequency of 50 Hz resulted in 80% of the maximal isometric force^11,26^. Knee extensor torques were measured with a strain bar (Entran Sensors & Electronics, USA) attached to the distal part of the tibia while the femur was rigidly fixed to prevent articular surface movement (<0.5 µm)^9,11^.

#### Dynamic cyclic loading

In loaded animals, stimulation trains of 0.5 s at 50 Hz every 4 s for 15 repeat contractions were applied to the quadriceps muscles every 30 minutes up to 240 minutes of observation. These contractions produced a compressive load at the knee articular surfaces corresponding to ~80% of the maximal possible muscle-induced joint compression. Multi-photon scans were taken before loading, and every 30 minutes up to 240 minutes following each bout of muscular loading (Fig. 2).

### Detection of apoptosis/necrosis in injured cartilage

Three animals representing the groups LMI, LMI and LMI were used for distinguishing between apoptosis and necrosis of chondrocytes. Mice were prepared as described under animal preparation section. Apoptosis/necrosis was detected using an apoptosis/necrosis detection kit (ab176750, abcam, Cambridge, UK) at a concentration of 2 µM of Apopxin Deep Red (apoptotic indicator) and 1 µM of Nuclear Green (necrotic indicator). Mice knees were incubated in the Apopxin Deep Red and Nuclear Green solution for 45 minutes in complete darkness prior to testing. After staining, knees were rinsed in phosphate-buffered saline (PBS) for 15 minutes, then mounted on the stage of a multi-photon laser scanning microscope (FVMPE-RS, Olympus, Japan). Two multi-photon lasers tuned to 1230 nm and 800 nm were used to excite the apoptotic and necrotic cell indicators respectively. Cells were visualized using the filter cubes Cy5 (Em = 660 nm) and FITC (Em = 520 nm) for apoptotic and necrotic cells respectively.

### Multi-photon and SHG image analysis

Prior to starting the muscular loading protocol and at intervals of 30 min, a simultaneous stack of images of collagen tissue along with live/dead cells, or a stack of apoptotic/necrotic cells was acquired (Fig. 2). A stack consisted of serial images of 1 µm thickness, ranging in depth of 250–350 µm from the medial side towards the middle of the joint. The field of view was 509 × 509 μm^2^ (pixel size: 0.994 µm × 0.994 µm; pixel dwell time: 2 µs; frame scan time: 1.084 s). Three-dimensional shapes of these stacks were reconstructed using open source software (ImageJ, NIH, USA). Live and dead cells were counted manually over the entire 3D volume. For knees containing cartilage injury, live and dead cell counts were discretized to intervals from 0–100 µm, 100–200 µm etc. up to a distance of 500 µm perpendicular to both sides of the injury (Fig. 1b). The percentage of cell death was calculated as C/C_0_ × 100%, where C is the number of dead cells and C_0_ is the total number of cells.

### Statistical analysis

Statistical analyses were made using SPSS software (Version 23.0, SPSS, Inc, Chicago, IL). Assumption of normality (Shapiro-Wilk test) and sphericity (Mauchley test) were tested for all dependent variables. If the assumption of sphericity was violated, the corrected value for non-sphericity with Greenhouse-Geisser epsilon was reported.

To determine the effect of meniscectomy, joint load, and cartilage injury on chondrocyte death, a one way analysis of variance (ANOVA) with repeated measures for 9 time points (0, 30, …, 240 minutes) and independent samples for 6 groups (LMI, LMI, LMI, LMI, LMI, LMI) was performed. Since the results showed a significant interaction effect for groups * times, a one-way ANOVA with Tukey corrections was used to compare the effects of different interventions over the time course of measurement. Significance was defined as p < 0.05.

## Results

**Figure 3:**
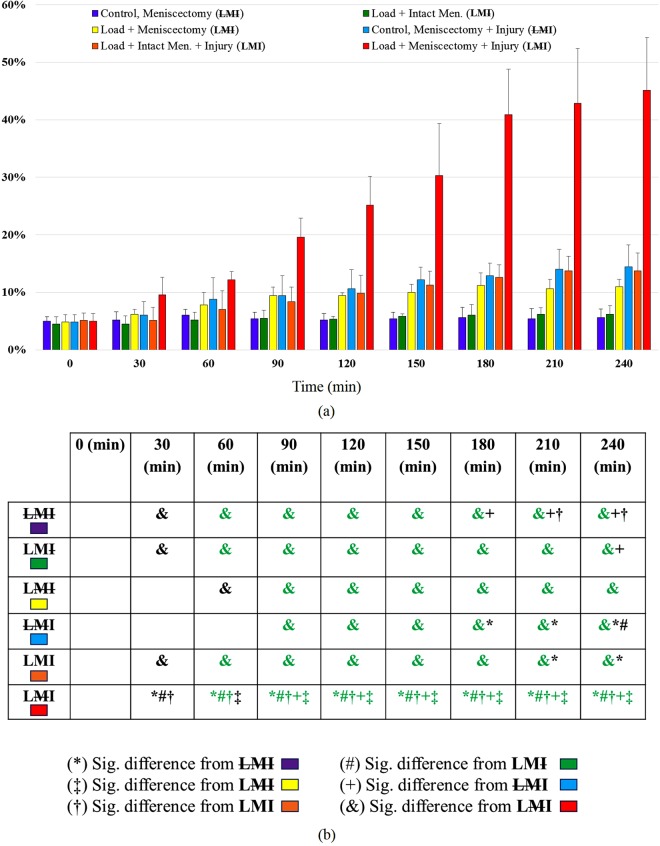
(**a**) Mean percentage of dead chondrocytes as a function of time in the six different groups; 2 non-loaded control groups LMI, LMI and 4 loaded groups LMI, LMI, LMI, LMI), Bars = SD. (**b**) Significant difference in cell death, green (P < 0.001), black (P ≤ 0.05).

**Figure 4:**
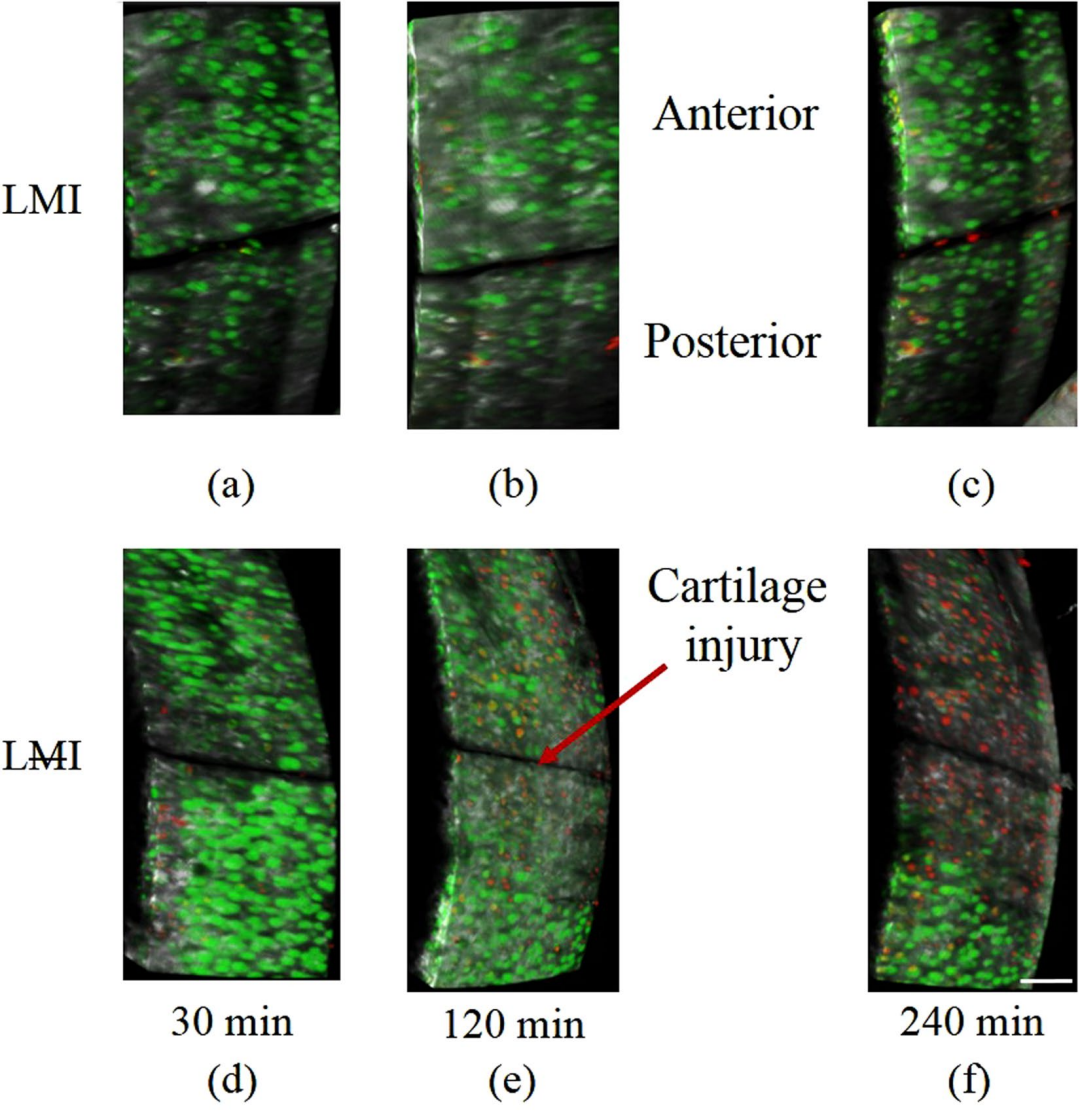
3D image of the cartilage showing the cartilage injury (~20 µm width, ~350 µm length), live (green), dead (red) cells and collagen tissue (grey) for three different times. The intact menisci prevent cell death in LMI group (**a**–**c**) while cell death increased rapidly for the corresponding conditions with the meniscectomy in LMI animal group (**d**–**f**). Bar = 80 µm.

**Figure 5:**
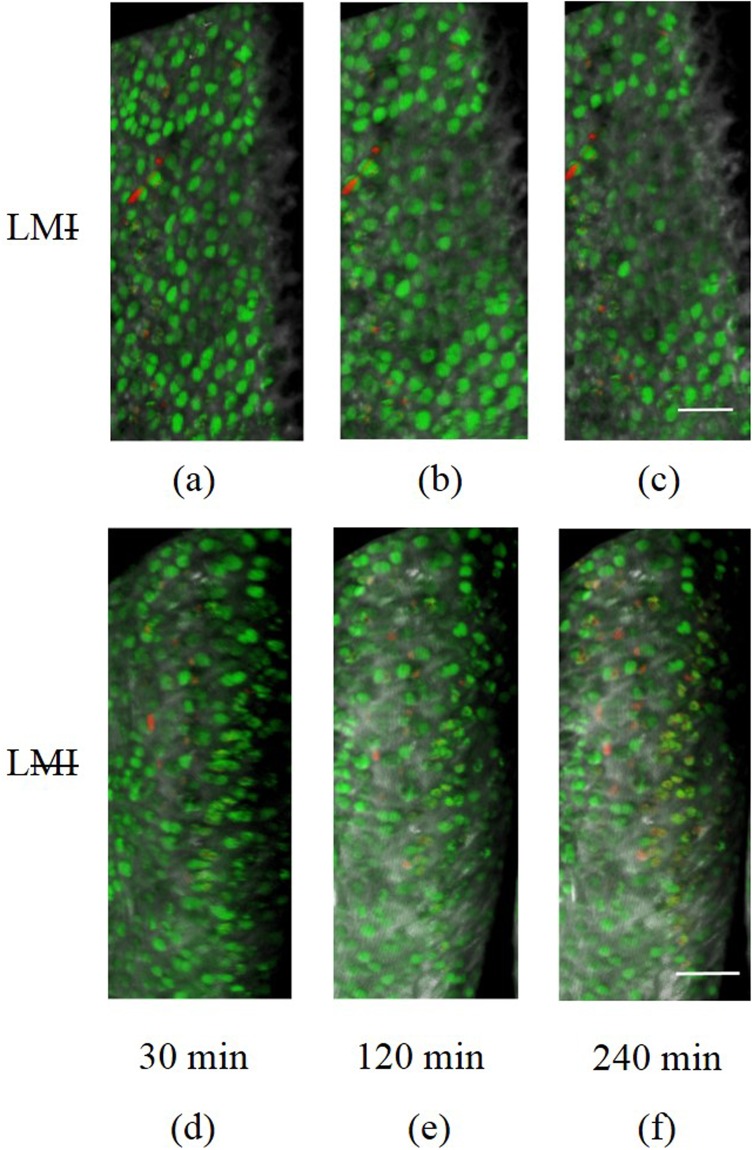
3D image of the cartilage showing live cells (green), dead cells (red), and collagen tissue (grey) for three time points in the protocol. The intact menisci prevent cell death in the LMI group (**a**–**c**) while cell death increased uniformly across the cartilage surface for the corresponding conditions in the LMI animal group (**d**–**f**). At 240 min, cell death approximately doubled in the LMI joints compared to the LMI joints. Bar = 60 µm.

Experiments were conducted to determine the role of the menisci under physiological magnitudes of joint loading on injured and uninjured cartilage surfaces. There were significant differences amongst groups as a result of applying joint loading, removing the meniscus, and introducing a focal cartilage injury. ANOVA demonstarated a significant interaction of groups * times for the percentage of dead cells (F_40,240_ = 25.13, p < 0.001) with increasing cell death associated with increased time and increased numbers of joint loading cycles. In the meniscus intact joint and focal cartilage injury group, chondrocyte death was significantly reduced compared to the meniscectomized joints following muscular loading (LMI vs LMI; 14 ± 4% vs 45 ± 9% at 240 min; Figs 3 and 4). Cell death started to increase significantly (p < 0.001) in the LMI group (meniscectomy with cartilage injury) at 90 min (corresponding to 45 isometric muscular loading cycles), resulting in rapid increases in cell death that reached 45% in 4 hours (Figs 3 and 4). For the same conditions, but with the meniscus intact (LMI group), cell death only became significant (p = 0.05) relative to control animals (LMI) at the 210 min time point, and at that time was similar to non-loaded meniscectomized knees (LMI vs LMI; 13.5 ± 4% vs 14.5 ± 4%; Fig. 3). Cell death was similar for the non-loaded, meniscectomized, non-injured group (LMI), and the loading protocol (LMI) groups at all time points. Cell death in (LMI) group was approximately half compared to the meniscectomized, uninjured (LMI) groups (Figs 3 and 5).

**Figure 6:**
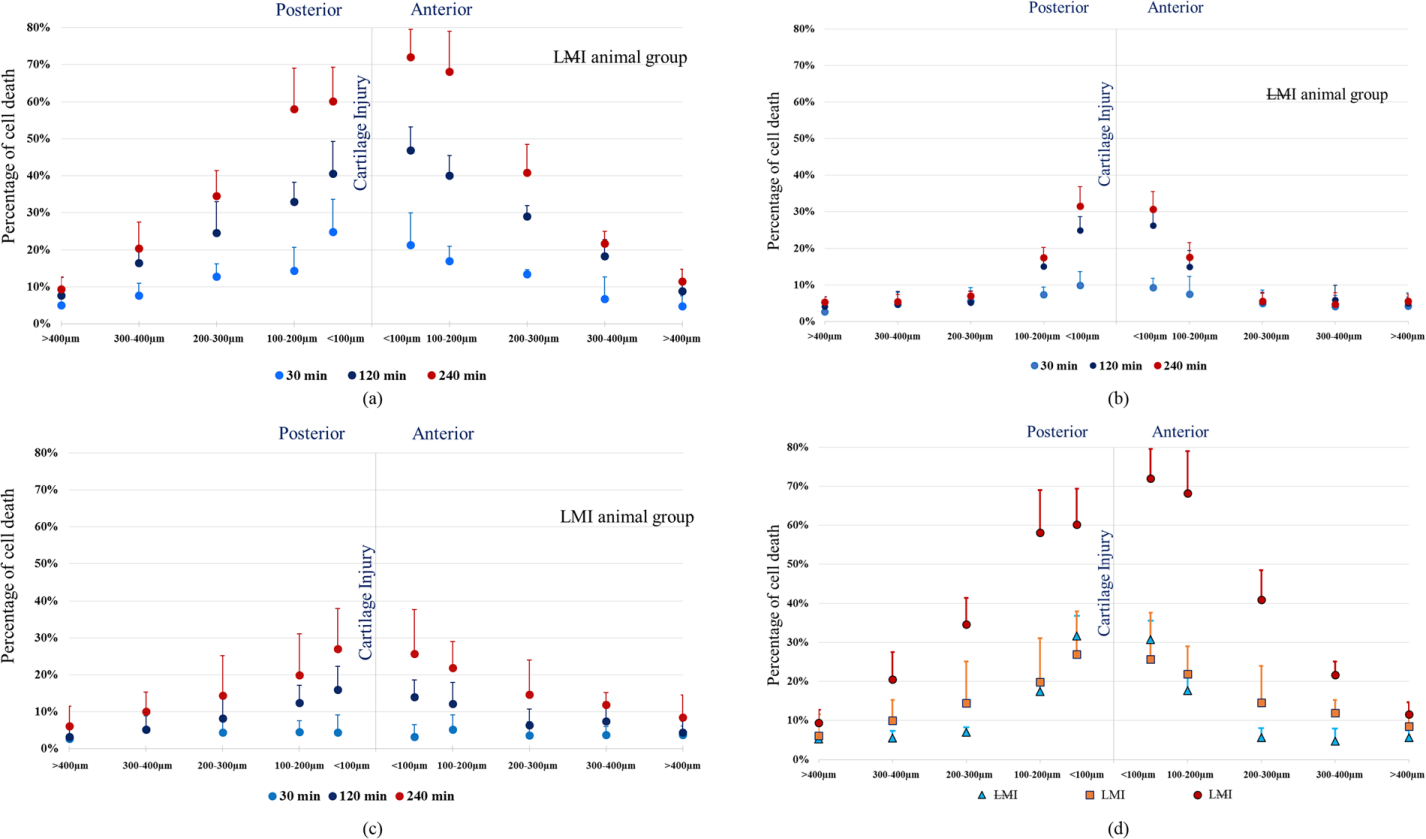
Mean percentage of dead chondrocytes as a function of distance from the cartilage injury (middle line) for three different groups (**a**) LMI; n = 8, (**b**) LMI; n = 8 and (**c**) LMI; n = 8 at three different times. (**d**) Percentage of cell death for the three groups LMI, LMI and LMI at 240 min. Bars = SD.

The percentage of cell death increased rapidly as a function of time near the cartilage injury (<200 µm on both sides of the injury) to reach >70% in the LMI group compared to ~ 30% for the corresponding condition with the meniscus intact (LMI animal group; Fig. 6). Cell death tended to be greater on the anterior compared to the posterior side of the injury in the LMI group, but this effect did not reach statistical significance (P = 0.056) (Fig. 6a). In the presence of injury without muscular loading (LMI group), cell death was concentrated near the cartilage injury (Fig. 6b). However, with the menisci intact and the joint loaded, cell death was virtually symmetrical relative to the cartilage injury (LMI animals; Fig. 6c). The distribution of cell death was similar between the non-loaded knees with injury and the loaded group when the meniscus was intact (Fig. 6d).

**Figure 7:**
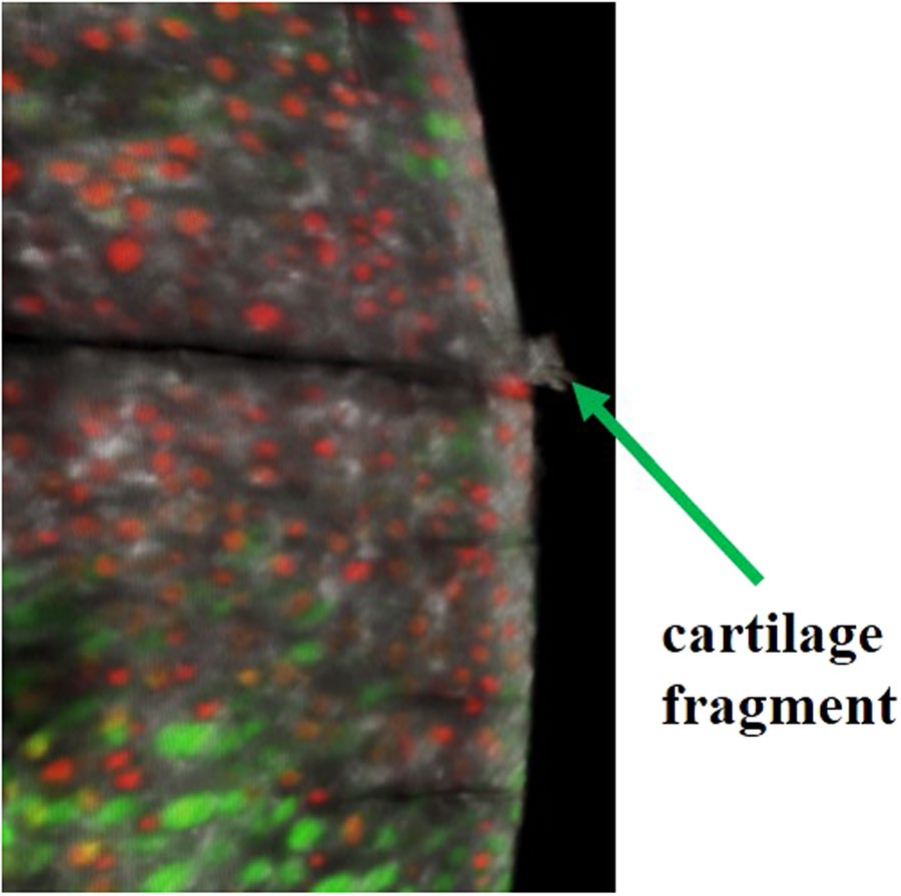
Release of cartilage fragments appeared clearly on the anterior side of the cartilage injury at 240 min or 120 contractions when the medial meniscus was removed (LMI animal group).

In the presence of cartilage injury, the meniscus helped to reduce cell death and prevent damage to collagen fibrils and the inegrity of the collagen fibrillar network (Fig. 4). Release of cartilage fragments was observed near the anterior side of the injury at 240 min or 120 muscular contractions (Fig. 7), while such fragments were never observed when the medial meniscus was left intact.

**Figure 8:**
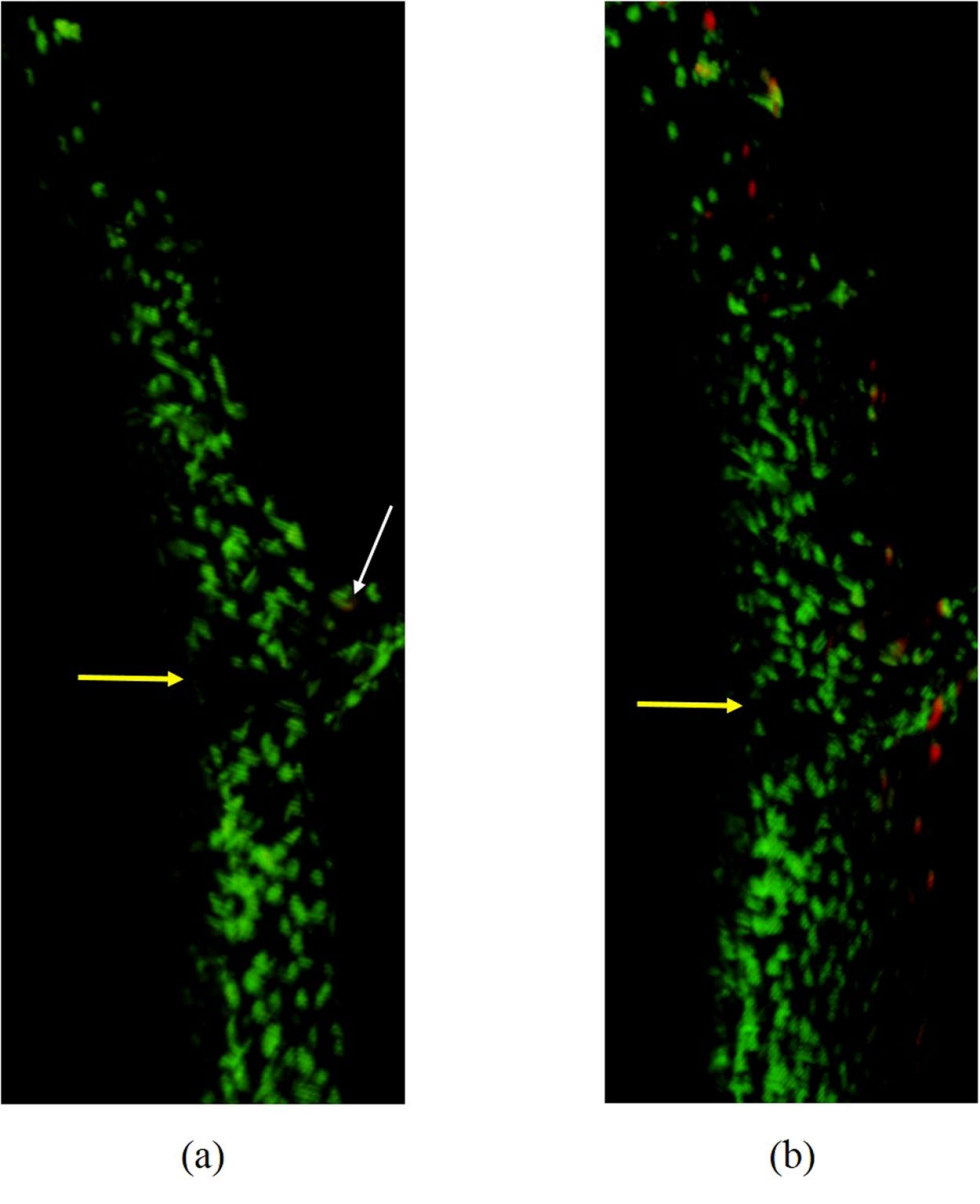
3D image of the cartilage showing necrotic (green) and apoptotic (red) cells at two time points (**a**) 120 min and (**b**) 240 min. Both images were taken from the LMI animal group. A single apoptotic cell was seen at 120 min (white arrow) near the cartilage injury (yellow arrow). Apoptotic cells start to progress from near the cartilage injury towards regions away from the injury at 240 min.

Necrosis was the dominant mechanism of cell death in this model. Cell necrosis began to develop in the four loaded animal groups LMI, LMI, LMI and LMI starting at 30 min. Apoptotic cells were observed only in the LMI group; one apoptotic cell was detected near the cartilage injury at 120 min. Apoptotic cells were identified close to and at a distance from the cartilage injury at 240 min (Fig. 8).

## Discussion

The primary result of this study is the quantitative demonstration of a profound, acute protective role of the menisci. Specifically, the menisci were found to reduce/prevent chondrocyte death and acute damage to the collagen fibrillar network in the loaded and/or injured mouse knee. It has been shown that cell death is correlated with the progression of OA, and that cell death plays an important role in the development of OA^27–30^. By reducing cell death and collagen damage, the menisci help maintain the overall health of articular cartilage, thereby possibly delaying or preventing the development of OA in the knee joint.

### Role of the Menisci in the Uninjured Joint

A large body of research has used meniscectomy or a DMM in otherwise uninjured joints to induce osteoarthritis in animal models^16,17,19^. Joints rapidly progress toward OA after the menisci are removed or de-functioned in these experimental models. Moreover, in the case of isolated meniscal tears in the human knee, partial or total meniscectomy drastically increases the risk of early onset OA^20,23^. In our study, the presence of the menisci in the murine knee helped to reduce the percentage of cell death in joints with intact cartilage. Cell death was approximately double in the meniscectomized, uninjured joint (LMI) compared to joints with an intact meniscus exposed to physiological loading conditions (LMI) (Figs 3 and 5), while the loaded intact meniscus group animals (LMI) had approximately the same level of cell death as the unloaded control group joints (LMI). These findings indicate that in a meniscectomized, but otherwise healthy joint, physiological muscular loading can result in increased chondrocyte death in an acute setting (4 hours).

Mechanically, the menisci are thought to play important roles in load distribution and joint stability^31,32^. Following meniscectomy, the tibiofemoral contact area decreases by (30–70%)^33^, resulting in increased contact stresses. Furthermore, it has been theorized that the initial loading of the menisci pre-stresses the articular cartilage, via fluid pressurization; preparing it for loading^24^. These two mechanisms combined could lead to significantly increased strains in the cartilage matrix in the meniscectomized knee. Since the predominant mechanism of cell death was necrosis in the area of cartilage-cartilage contact^11^, it is probable that overloading (excessive strain) caused the cell death.

### Role of the Menisci in the Injured Joint

In the presence of a focal cartilage defect, almost 50% of the chondrocytes were dead after 4 hours in the loaded, meniscectomized knee (LMI), while only 14% of the cells were dead for the corresponding conditions with the menisci intact (LMI). The rapid cell death in meniscectomized knees is in agreement with previous findings. Bartell *et al*.^34^ found that chondrocyte death was highly correlated with a threshold of 8% cartilage strain, and chondrocyte death developed within 2 h of load application in normal, neonatal bovine cartilage explant samples. We reported recently that our muscular loading protocol produces articular cartilage strains averaging 10% in murine knee joints^11^.

Cartilage injury has been shown to be associated with altered geometry and decreased joint stability^32,35^. A cartilage defect typically results in stress concentrations near the defect site, and a corresponding increase in local matrix strain^36^. Cartilage defects typically also result in an increase in local permeability, reducing cartilage stiffness under rapidly applied load conditions, thereby exposing cells to potentially larger strains than they would experience in the intact cartilage. Like for the uninjured joints, necrosis was also the main mechanism of cell death in the injured knees, indicating that mechanical insult is the likely cause of the observed cell death. However, the amount of cell death was significantly elevated in the meniscectomized knees in the injured group compared to the uninjured group (LMI vs LMI). Increased local strains caused by the cartilage injury, and the increased contact stresses in the absence of the menisci, may explain this finding. This finding is consistent with the work of Peña and coauthors who used finite element analysis to evaluate joint stresses and strains in the medial femoral condyle containing lesions^37^. These authors predicted increased cartilage strains adjacent to a defect, and the effects of focal defects in a load-bearing region were more pronounced than in a non-load-bearing region. The menisci decreased the local stresses, thereby protecting the cells adjacent to the defect from injurious strains. This mechanism seems plausible based on our results, as the total cell death in our LMI and LMI animals were not significantly different, demonstrating that muscular loading is well accommodated in an injured joint that contains menisci.

Cell death was highest near the cartilage injury. Cell death in the (LMI) group reached >70% within an area of ±100 µm from the cartilage injury, decreasing to ~ 40% between 200–300 µm from the injury site (Fig. 6a,d). With muscular loading and the meniscus intact (LMI), cell death within ±100 µm is (~30%) similar to that found in the unloaded surgical control group samples (LMI), decreasing to <15% between 200–300 µm from the injury site (Fig. 6b–d). The menisci are known to distribute stress across the articulating surfaces of the tibia and femur, and are thought to mediate fluid flow in the articular cartilage^24^. A more even stress distribution appears to protect chondrocytes from necrosis potentially caused by excessive cell membrane strains.

The meniscectomized knees (LMI) were the only ones to show detectable apoptotic cell death near the areas of collagen damage at 240 minutes. Data presented in another study^38^ demonstrated that mechanical injury induces chondrocytes death in the form of apoptosis in bovine and human explants, and the rate of apoptosis increased 3 hours post injury. We do not know what triggered apoptosis, but speculate that this may have been a function of an inflammatory cascade initiated by chondrocyte necrosis and associated upregulation of caspases^39^. Chondrocytes may also initiate apoptosis when losing their natural ECM attachment resulting from collagen disruption^39^. Chondrocyte apoptosis has been observed in osteoarthritic cartilage and in articular cartilage explants injured by surgical excision and cyclic compression^40–44^. The combined loss of chondrocytes due to necrosis and apoptosis in the LMI condition resulted in a profound hypo-cellularity that is also observed in osteoarthritis^45,46^.

Extreme care was taken during the meniscectomy not to injure the joint surfaces and avoid any bleeding into the joint space. All animals with joint bleeding were discarded from analysis in order to eliminate any artefactual cell death scenarios. Despite performing all surgical interventions with great care, there are several limitations that should be kept in mind when interpreting our results. First, the use of a mouse model may have limited translational fidelity to human meniscus injury. However, this model allows us to control the loading regime and image the time course of changes in cell viability in real time with high spatial resolution that cannot be achieved in human studies at this time. Furthermore, the focal defect induced with a scalpel is not representative of a focal injury found in early joint degeneration. This experimental condition represents a best-case scenario, where the defect is not chronic and not the result of degenerative changes. Yet, even in this ideal scenario with an otherwise healthy cartilage, meniscectomy resulted in significant cell death and cartilage degeneration in a matter of hours when samples were subjected to muscular joint loading. We observed the mice for 4 hours after the induction of the injury, therefore any long-term health outcomes remain unknown. However, the DMM model has been used to demonstrate progression of OA to a Kellgren Lawerence Grade V by 8 weeks post-meniscectomy in rodents^17^. Thus, one might expect a similar long-term fate for the animals of our study, had they been observed for a sufficient period of time.

Another limitation of the current study was that we could not measure the articular cartilage strains during muscular loading for the meniscus intact conditions. The meniscus covered and sealed the entire surface of the femoral condyle and prevented the multi-photon laser from penetrating the cartilage deformation site; thus imaging cartilage deformation was not possible.

### Significance

We provide first direct evidence that the menisci play an integral role in chondrocyte protection from necrosis in the intact and lesioned knee for acute, and physiologically relevant (muscular) loading conditions.
