# Publisher Correction: Menisci protect chondrocytes from load-induced injury

**DOI:** 10.1038/s41598-019-40293-3

**Published:** 2019-04-09

**Authors:** Z. Abusara, S. H. J. Andrews, M. Von Kossel, W. Herzog

**Affiliations:** Human Performance Laboratory, Faculty of Kinesiology, University of Calgary, Alberta, Canada

Correction to: *Scientific Reports* 10.1038/s41598-018-32503-1, published online 20 September 2018

The original version of this Article contained errors in the naming of the animal groups, where LMI, LMI, LMI, LMI, LMI and LMI were all written as LMI.

These errors have now been corrected in the HTML and PDF versions of this Article.

